# The fluid management of adults with severe malaria

**DOI:** 10.1186/s13054-014-0642-6

**Published:** 2014-11-21

**Authors:** Josh Hanson, Nicholas M Anstey, David Bihari, Nicholas J White, Nicholas P Day, Arjen M Dondorp

**Affiliations:** Menzies School of Health Research, Royal Darwin Hospital, Rocklands Drive, Tiwi, NT 0810 Australia; Mahidol-Oxford Tropical Medicine Research Unit, Faculty of Tropical Medicine, Mahidol University, 420/6 Rajvithi Road, Bangkok, 10400 Thailand; Prince of Wales Hospital, Barker Street, Randwick, NSW 2031 Australia; Centre for Tropical Medicine, Nuffield Department of Medicine, University of Oxford Roosevelt Drive, Oxford, OX3 7FZ UK

## Abstract

Fluid resuscitation has long been considered a key intervention in the treatment of adults with severe falciparum malaria. Profound hypovolemia is common in these patients and has the potential to exacerbate the acidosis and acute kidney injury that are independent predictors of death. However, new microvascular imaging techniques have shown that disease severity correlates more strongly with obstruction of the microcirculation by parasitized erythrocytes - a process termed sequestration. Fluid loading has little effect on sequestration and increases the risk of complications, particularly pulmonary edema, a condition that can develop suddenly and unpredictably and that is frequently fatal in this population. Accordingly, even if a patient is clinically hypovolemic, if there is an adequate blood pressure and urine output, there may be little advantage in infusing intravenous fluid beyond a maintenance rate of 1 to 2 mL/kg per hour. The optimal agent for fluid resuscitation remains uncertain; significant anemia requires blood transfusion, but colloid solutions may be associated with harm and should be avoided. The preferred crystalloid is unclear, although the use of balanced solutions requires investigation. There are fewer data to guide the fluid management of severe vivax and knowlesi malaria, although a similar conservative strategy would appear prudent.

**Electronic supplementary material:**

The online version of this article (doi:10.1186/s13054-014-0642-6) contains supplementary material, which is available to authorized users.

## Introduction

More than 10% of adults with severe falciparum malaria (SFM) requiring ICU admission will die from the disease [1]. As this population is almost universally hypovolemic [2,3] and the severity of lactic acidosis and the presence of acute kidney injury (AKI) on admission to the hospital are strong prognostic indicators [1,4], it has seemed intuitive that any volume deficit in these patients should be promptly corrected.

The implementation of guidelines that include early, aggressive fluid loading of patients with bacterial sepsis improves outcomes [5]. In the resource-poor setting, prompt fluid resuscitation is a life-saving therapy for conditions as diverse as dengue shock syndrome and cholera [6,7]. However, unlike these conditions, hypotension is relatively infrequent in SFM and is not necessarily associated with increased mortality [8]. Furthermore, fluid loading has a limited effect on the obstructed microvascular circulation which is central to disease pathogenesis [9], and as adults with SFM have a generalized increase in vascular permeability [10], fluid loading may lead to complications. In the pulmonary circulation, this can result in acute pulmonary edema (APO), which is often fatal in these patients [11].

As recently as 2010, World Health Organization (WHO) treatment guidelines observed that ‘in adults, there is a very thin dividing line between over-hydration, which may produce APO, and under-hydration contributing to shock, worsening acidosis and renal impairment’ [12]. Against this backdrop, the best approach for clinicians caring for patients has been unclear, resulting in lively debate [13,14]. However, recent studies in adults and children with SFM can now offer some guidance.

## Review

### Assessing volume status

The clinical assessment of a patient’s volume status is notoriously unreliable [15,16]. In SFM, it is even more challenging as physical signs of hypovolemia - including tachycardia, oliguria, and confusion - can result from the disease itself. In the PRISM (PiCCO-guided Resuscitation in Severe Malaria) study, in which 28 critically ill, hypovolemic Bangladeshi and Indian adults with SFM received fluid resuscitation guided by transpulmonary thermodilution, physical examination failed to identify patients who were either volume-responsive or at greater risk of APO [17]. Recognizing the limitations of the physical examination, the 2010 WHO treatment guidelines empirically recommended using a central venous catheter (CVC) in critically ill patients and a target central venous pressure (CVP) of 0 to 5 cm H_2_O [12]. However, in the Vietnamese Hemodynamic Study (VHS) of 24 adults with SFM who received fluid loading guided by pulmonary artery occlusion pressure (PAOP), there was no relationship between CVP and cardiac output either before or after fluid resuscitation, and almost half the patients with an admission CVP of not more than 0 cm H_2_O were not volume-responsive [18]. Similarly, in PRISM, there was no relationship between CVP and cardiac output before or after fluid resuscitation, and almost 40% of those patients with a CVP of not more than 0 cm H_2_O were not volume-responsive [19].

A CVP of between 0 and 5 cm H_2_O also failed to protect patients against APO: the majority of patients in PRISM and VHS with APO on enrollment had a CVP of not more than 5 cm H_2_O [18,19]. There were similar findings in a Thai series and in smaller series of imported malaria [20-22]. These observations and the additional risks of using CVCs have led the WHO to rescind their recommendation for CVP-guided fluid resuscitation [23]. This has coincided with a growing skepticism about the utility of the CVP to guide fluid resuscitation in other critically ill populations [24].

So where does this leave the clinician? Physical findings cannot be ignored, but if used to guide fluid resuscitation, they need to be interpreted cautiously and informed by repeated assessment, clinical judgment, and laboratory and radiological investigations. In one series, transpulmonary thermodilution appeared to be helpful as monitoring of extravascular lung water (EVLW) during fluid loading promptly identified incipient APO [25], but further study is required to define its optimal role.

### Contribution of volume status to clinical manifestations

Yet even if hypovolemia can be accurately identified, recent data suggest a relatively limited pathological role in adults with SFM. Every patient in PRISM was hypovolemic on admission when assessed with transpulmonary thermodilution, yet the more hypovolemic patients were actually less acidotic. Renal function was also unrelated to volume status on admission; indeed, after 24 hours of fluid resuscitation, the more hypovolemic patients had lower plasma creatinine concentrations [25]. In VHS, patients with lower systemic oxygen delivery (DO_2_) had neither greater acidosis nor renal impairment [18], whereas in a small Thai series, patients admitted with AKI had a normal blood volume [26].

**Figure 1:**
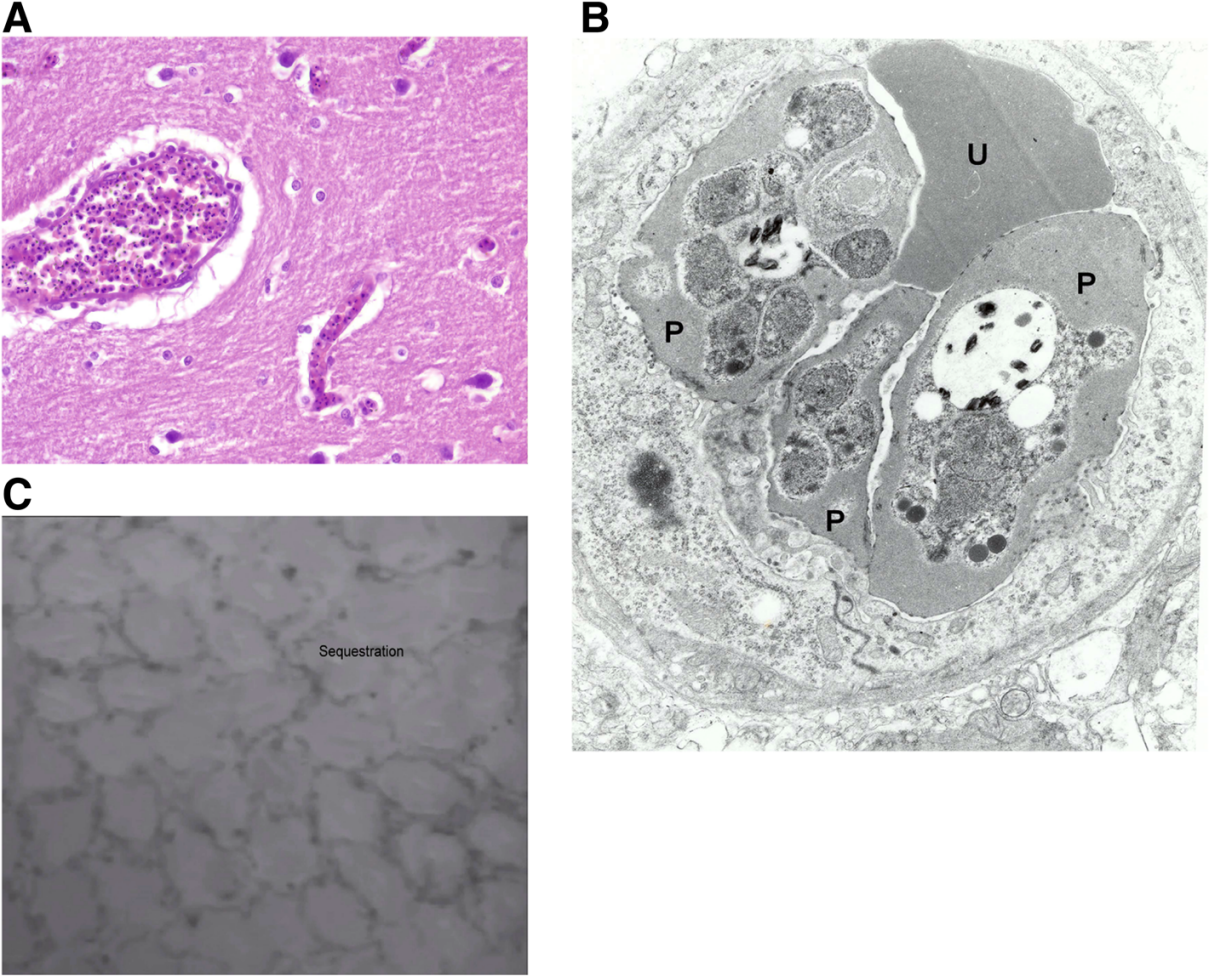
**The appearance of microvascular sequestration with the use of different imaging techniques. (A)** Photomicrograph of a brain section from an adult Vietnamese patient who died with cerebral malaria, demonstrating blood vessels packed with parasitized erythrocytes (hematoxylin-eosin stain, magnification × 400, courtesy Gareth Turner). **(B)** An electron micrograph of a capillary demonstrating microvascular obstruction. Parasitized erythrocytes (P) obstruct the passage of an uninfected erythrocyte (U) (courtesy of Emsri Pongponratn). **(C)** Still from an orthogonal polarization spectral imaging video (Additional file 1) of a patient with severe falciparum malaria, showing the cobblestone pattern of the capillaries surrounding the rectal crypts. The polarization filter causes red structures to appear dark grey, hence erythrocytes are visible as grey spots; their movement can be measured by using image-analysis software. The presence of sequestration is suggested in vessels where there is no erythrocyte movement.

Instead, it is the changes at a microvascular level, including sequestration, endothelial dysfunction, and changes in blood rheology, that appear more important [27] (Figure 1, Additional file 1). Sequestration - the process in which parasitized erythrocytes pack the microcirculation, avoiding clearance by the spleen but physically obstructing blood flow - correlated with plasma lactate concentrations before and after resuscitation in PRISM [25] and on admission in two other Bangladeshi series [28,29]. Meanwhile, autopsy studies have shown greater renovascular sequestration in SFM patients with AKI [30].

Conversely, features of SFM that might be attributed to fluid excess usually exist in the absence of volume overload. Overzealous fluid resuscitation will exacerbate APO, but in PRISM, 70% of patients were hypovolemic at the time it developed and the remainder were euvolemic [25]. Adults with cerebral malaria are universally hypovolemic at presentation; cerebral edema can occur, but this tends to be in the agonal setting as part of a multi-organ dysfunction syndrome [31].

### Effects of fluid resuscitation: acidosis and renal dysfunction

Two series - PRISM and VHS - examined the response of adults with SFM to fluid resuscitation in detail (Table 1) [18,25]. In PRISM, patients received a total of 81 fluid challenges (targeting a global end-diastolic volume index (GEDVI) of more than 680 mL/m^2^) but were volume-responsive (increase in cardiac index by more than 15%) on only 28% of these occasions; meanwhile, 50% of the patients in VHS were volume-responsive to their single fluid challenge (which targeted a PAOP of 9 to 12 mm Hg). There was no clinically meaningful increase in DO_2_ with fluid resuscitation in either study. More importantly, there was no significant improvement in acidosis, an important finding as acid–base status at admission was the strongest prognostic indicator in both series. Hyperchloremia from the chloride-rich fluid load partly explained these findings, but the most important explanation appears to be the limited effect of fluid resuscitation on microvascular sequestration, the principal cause of the acidosis. Despite 6 hours of liberal fluid loading in PRISM, 91% of patients still had visible sequestration and in 35% the degree of sequestration was unchanged or had increased. Sequestration was still visible in almost half of those assessed at 48 hours [25].

**Table 1:** **Response to fluid resuscitation in the two series to specifically examine the issue in adults with severe falciparum malaria**

	**VHS** ^**a**^ [18]			**PRISM** ^**b**^ [25]		
	**Baseline**	**Post-resuscitation** ^**c**^	**Change**	**Baseline**	**Post-resuscitation** ^**c**^	**Change**
Plasma bicarbonate, mmol/L	13.9	12.8	−0.1	16.9	12.1	−1.9
	(10.5 to 17)	(11.2 to 17.3)	(−1 to 1)	(13.8 to 17.8)	(13.7 to 15.4)	(−3.7 to −0.6)
Plasma lactate, mmol/L	6.3	6.3	−0.1	3.2	1.7	−1
	(2.7 to 9)	(2.7 to 9)	(−0.6 to 0.4)	(1.9 to 5.4)	(1.4 to 3.1)	(−1.8 to 0.3)
pH^d^	7.36	7.35	−0.01	7.34	7.31	−0.05
	(7.29 to 7.41)	(7.29 to 7.39)	(−0.01 to 0.01)	(7.32 to 7.37)	(7.24 to 7.34)	(−0.1 to 0.1)
Base deficit, mEq/L	11	13	0.6	9	13	3
	(7 to 13)	(6 to 14)	(0.3 to 1.8)	(8 to 12)	(10 to 14)	(0 to 5)
Strong ion gap, mEq/L^e^	-	-	-	11.2	12.4	1.2
				(7.2 to 15.2)	(9.4 to 15.2)	(−2.1 to 3.6)
CVP, cm H_2_O	2	5	3	5	10	6
	(0 to 4)	(3 to 6)	(0 to 6)	(−2 to 8)	(7 to 14)	(1 to 10)
PAOP, mm Hg^f^	6	11	5	-	-	-
	(4 to 7)	(8 to 12)	(2 to 8)			
Heart rate, beats per minute	115	111	−6	101	99	0
	(100 to 130)	(94 to 119)	(−11 to 0)	(93 to 112)	(90 to 110)	(−11 to 9)
Glasgow Coma Scale score^g^	8	-	-	12	14	0
	(6 to 13)			(8 to 15)	(8 to 15)	(0 to 2)
MAP, mm Hg	79	79	1	88	94	7
	(71 to 85)	(74 to 89)	(−2 to 7)	(79 to 98)	(82 to 108)	(−2 to 17)
Cardiac index, L/min per m^2^	4.06	5	0.63	3.08	3.64	0.49
	(3.23 to 4.82)	(3.8 to 5.49)	(0.15 to 1.06)	(2.84 to 3.28)	(3.38 to 4.13)	(0.18 to 1.13)
DO_2_I, mL/min per m^2^	454	470	18	421	403	15
	(341 to 557)	(371 to 578)	(−21 to 87)	(348 to 482)	(331 to 532)	(−90 to 86)
SVRI, dynes-sec/cm^5^ per m^2^	1,430	1,174	−139	2,155	1,926	−203
	(1,193 to 2,039)	(945 to 1,874)	(−434 to −25)	(1,779 to 2,532)	(1,552 to 2,320)	(−751 to 170)
Hemoglobin, g/dL	8.8	7.4	−0.7	10.4	9.1	−1.6
	7.2 to 10.8	(6.1 to 9.7)	(−1.6 to −0.1)	(8.5 to 12.6)	(7.1 to 10.5)	(−2.5 to −0.2)
SaO_2_/FiO_2_ ratio	436	484	0	455	443	−9
	(374 to 575)	(360 to 552)	(−25 to 25)	(306 to 467)	(271 to 462)	(−23 to 2)
GEDVI, mL/m^2e^	-	-	-	472	585	91
				(429 to 571)	(539 to 638)	(10 to 126)
EVLW, mL/kg^e^	-	-	-	8	10	1
				(6 to 9)	(8 to 11)	(1 to 3)
PVPI^e^	-	-	-	2.29	2.25	0.05
				(1.82 to 2.45)	(1.97 to 2.6)	(−0.16 to 0.32)
Plasma creatinine, μmol/L^g^	339	-	-	158	132	−26
	(220 to 572)			(106 to 255)	(88 to 211)	(−62 to 18)

Values are presented as the median (interquartile range). ^a^Median (range) of 3,230 mL (1,035 to 7,000) over the initial 6 hours and 5,450 mL (1,985 to 13,720) over the first 24 hours. ^b^Median (range) fluid bolus of 918 mL (350 to 2,000) was delivered over a median (range) of 75 minutes (30 to 225). ^c^Post-initial resuscitation in the Vietnamese Hemodynamic Study (VHS), post-6 hours of fluid resuscitation in PiCCO-guided Resuscitation in Severe Malaria (PRISM) except plasma creatinine which was assessed after 24 hours. ^d^pH: arterial in VHS, central venous in PRISM. ^e^Not measured in VHS. ^f^Not measured in PRISM. ^g^As fluid load occurred over a median of only 75 minutes in VHS, Glasgow Coma Scale score and plasma creatinine were not repeated immediately post-resuscitation. CVP, central venous pressure; DO_2_I, oxygen delivery; EVLW, extravascular lung water; GEDVI, global end-diastolic volume index; MAP, mean arterial pressure; PAOP, pulmonary artery occlusion pressure; PVPI, pulmonary vascular permeability index; SaO_2_/FiO_2_, oxygen saturation (percentage)/fraction of inspired oxygen; SVRI, systemic vascular resistance index.

The persisting acidosis also resulted from the fluid load’s failure to significantly improve renal function. In PRISM, no anuric patient regained a urine output with fluid loading and 75% later required renal replacement therapy (RRT). In the patients with significant renal impairment still passing urine, 50% had an increase in plasma creatinine despite 24 hours of fluid resuscitation. In VHS, renal function was as likely to deteriorate in the patients receiving a fluid bolus as in those who did not [18].

In other series, the strongest predictor of a salutary renal response to fluid resuscitation was the renal function on admission. Only 5% of anuric patients regained a urine output with fluid loading and diuretics in a Vietnamese series [32]. In a series of Bangladeshi patients receiving fluid resuscitation guided by clinical assessment, two thirds with significant renal impairment later required RRT [33]. Patients with milder renal impairment are more likely to respond, but kidney function can still deteriorate. In the Bangladeshi series, 7.4% with a normal plasma creatinine on admission later required RRT [33]. In an Indian series, 11% of those without renal failure on admission later developed the complication [34]. Renal tubular injury is proportional to the duration and extent of renal hypoperfusion, and the failure of fluid resuscitation to improve renal function may be due to delays in its initiation. However, sequestration also leads to tubular hypoperfusion, and its persistence despite fluid and anti-malarial therapy [25,35] may again explain why AKI continues to evolve in some patients.

### Pulmonary edema and cerebral malaria

In PRISM, 74% of patients developed clinical or volumetric (EVLW of at least 10 mL/kg) evidence of APO during resuscitation. All patients were hypovolemic or euvolemic (normal GEDVI) at the point that APO developed [25]. Fluid loading was more conservative in VHS, and clinical APO occurred less frequently [18]. These findings suggest that increased pulmonary vascular permeability is common in adults with SFM and that the risk of APO is increased by liberal fluid loading.

There is little pathological or radiological evidence that vasogenic edema plays a major role in the pathogenesis of cerebral malaria [31,36], a principle supported by an absence of clinical evidence of cerebral edema in any of the patients in PRISM [25] and the absence of neurological deterioration in any of the patients receiving fluid loading in VHS [18].

### Other clinical findings

After 24 hours of fluid resuscitation, almost 90% of patients in the PRISM study were still hypovolemic and most had developed marked generalized peripheral edema. The patients with marked edema were more likely to develop APO and were more likely to die. Notably, in the first 24 hours, there was a median fall in the intravascular volume of the patients who later died compared with a median increase in survivors [25]. The mechanism for this generalized edema is uncertain, although endothelial activation and dysfunction probably contribute. Plasma angiopoietin-2 concentrations correlate with outcome and disease severity in adults with malaria [37]. Endothelial activation and sequestration may also disrupt the endothelial glycocalyx, diminishing vascular integrity [38].

### Role of intensive care unit support

However, despite the documented harm of fluid resuscitation, survival in PRISM and VHS was better than expected when controlled for disease severity [4]. This was particularly notable in VHS, in which patients received relatively inferior anti-malarial therapy (quinine or artemether). Rather than suggesting any occult benefit of fluid resuscitation, this more likely reflects the two studies’ ICU setting which facilitated access to supportive care and well-trained clinicians. In the well-resourced ICU, mortality rates of adults with SFM are much lower [1,39].

Resuscitation strategies should also be judged by the ease with which their complications can be remedied. It may be better to err on the side of under-hydrating adults with SFM as APO can occur suddenly and unpredictably and is frequently fatal even if mechanical ventilation is available. Conversely, AKI develops less abruptly and has a greater therapeutic window, allowing referral to nephrology services, where it has a better response to treatment.

Despite the availability of mechanical ventilation, 89% of Indian patients with an arterial partial pressure of oxygen/fraction of inspired oxygen (PaO_2_/FiO_2_) ratio of less than 200 mm Hg died in one large series [34] and 70% of Thai patients with a PaO_2_/FiO_2_ ratio of less than 100 mm Hg died in another [22]. Over 60% of South African patients with APO died despite mechanical ventilation [40], and even in European national referral hospitals, patients die from malaria-related APO [39]. Conversely, in patients with AKI requiring RRT, mortality fell from 75% to 26% with a mean duration of 3 days of peritoneal dialysis (PD) [32]. When hemofiltration was compared with PD in Vietnamese patients with AKI and severe infection (69% of whom had SFM), patients receiving hemofiltration had a mortality of only 15% and a faster resolution of acidosis and required RRT for fewer days [41].

### Multi-organ dysfunction syndrome

Although there is a tendency to categorize adults with SFM as either ‘dry’ and at increased risk of acidosis and AKI or ‘wet’ and at increased risk of APO, this appears too simplistic an approach. Multi-organ involvement is the rule in the patients with the most severe disease. Fluid management is challenging in these patients, although again a more conservative approach is more likely to be beneficial [42].

In one Indian SFM series, 69% of adults with both AKI and APO died, the case-fatality rate was 14% if APO occurred in isolation, and there were no deaths in patients with only AKI [43]. In another Indian study, the risk of death increased with both renal failure and respiratory failure, but all patients with AKI alone survived and no patient in the series had APO in isolation [34]. In a South African series, 7 out of 8 deaths occurred in patients with concomitant AKI and APO [40], whereas in a French series, most patients with APO had multi-organ involvement (83% had AKI) and were more likely to die [44]. In a Portuguese series, over three quarters of the deaths occurred in patients with concurrent AKI and APO [45], and in PRISM all of the patients who died had APO while anuric [25]. In a British series, the only patient whose death was directly attributable to malaria died of multi-organ failure with concomitant AKI and APO [39].

### Lessons from pediatric malaria

SFM has a different clinical spectrum in adults and children: in adults, coma duration is longer, severe anemia is less frequent, and AKI and APO are much more common [46]. However, there are important similarities in pathophysiology in the two populations, particularly the fundamental role of sequestration. In studies examining the fluid resuscitation of adults and children with SFM, there are many comparable findings.

Although hypovolemia is present in children with SFM, it is relatively mild when assessed by using standardized measures and is not commensurate with the degree of end-organ pathology. Gabonese children with SFM were only mildly dehydrated when assessed isotopically; despite this, over half had a plasma lactate of more than 5 mmol/L [47]. Retinal changes consistent with sequestration have been documented in children with SFM and correlate with outcome and sequestration seen at autopsy [48-50]. These findings suggest that, as in adults, sequestration and endothelial dysfunction can explain the clinical manifestations of disease in children without hypovolemia being invoked.

The fluid resuscitation of children with SFM has been explored on a larger scale than in adults, culminating in the landmark FEAST (Fluid Expansion as Supportive Therapy) study [51]. Here, febrile African children with clinical evidence of hypoperfusion (57% with *P. falciparum* parasitemia) were randomly assigned on admission to the hospital to receive boluses of either 20 to 40 mL/kg of 5% albumin or 0.9% saline or to receive no boluses; standard care was otherwise provided. The mortality was increased to a similar degree in the children receiving an albumin or saline bolus (relative risk of death: 1.59) compared with the control group.

A retrospective analysis suggested that the increased mortality resulted from cardiovascular collapse rather than fluid overload *per se*, although this conclusion was somewhat hampered by limited data regarding the participants’ deaths. The mechanism for this cardiovascular collapse was unclear, although a rapid reduction in sympathetically mediated compensatory mechanisms and treatment-induced hyperchloremic metabolic acidosis were suggested. Although APO and increased intracranial pressure were suspected in some patients, neither was identified frequently [52].

### Lessons from the critical care literature

The hazards of fluid resuscitation are well recognized, and fluid excess has been linked to increased mortality in several critically ill adult populations [53-57]. Various hypotheses have been proposed to explain these findings. Up to 80% of a crystalloid bolus leaves the intravascular space of volunteers within an hour of its administration [58,59], and this figure may be higher in critically ill adults in whom increased capillary permeability is common [60]. The resulting tissue edema might lead to organ dysfunction by obstructing capillary and lymphatic flow and impairing oxygen and metabolite diffusion [61,62].

Liberal fluid resuscitation is one of the central, albeit controversial, components of the Surviving Sepsis Guidelines [63,64]. Although adherence to the guidelines improves outcomes [65], assessing the contribution of fluid resuscitation is complicated by the fact that it is delivered as one component of a bundle of care including antibiotics, vasopressors, and expert medical and nursing care; an independent benefit of fluid resuscitation was not identified in several studies [66,67]. Meanwhile, in patients with acute lung injury, a restrictive fluid strategy has been shown to improve oxygenation without an increased incidence of shock or RRT requirement [42].

Even if a patient is fluid-responsive, simply increasing DO_2_ is not necessarily associated with improved outcomes [68]. If there is an adequate cardiac output and mean arterial pressure - as was the case in the majority of patients in PRISM and VHS even prior to fluid loading - what are we expecting any extra administered fluid to do?

### Optimal resuscitation fluid

The WHO recommends 0.9% saline to rehydrate adults with malaria, although recent concerns about the association between the use of chloride-rich fluids and acidosis and AKI may be relevant [69]. Although balanced solutions have shown survival benefit in some ICU populations [70], they have yet to be trialed in malaria. Few clinical data support the use of colloids in critically ill populations [71,72]. The fact that some preparations are associated with an increased risk of AKI and bleeding is concerning given the incidence of these complications in SFM. Against this background, the current WHO recommendation to treat shocked patients with plasma expanders may need revision [23]. As children receiving albumin in the FEAST study had an increased mortality, it cannot be recommended in adults with malaria [51]. The WHO empirically recommends a threshold for transfusion of 7 g/dL in adults with SFM.

### Suggested fluid resuscitation strategy

**Figure 2:**
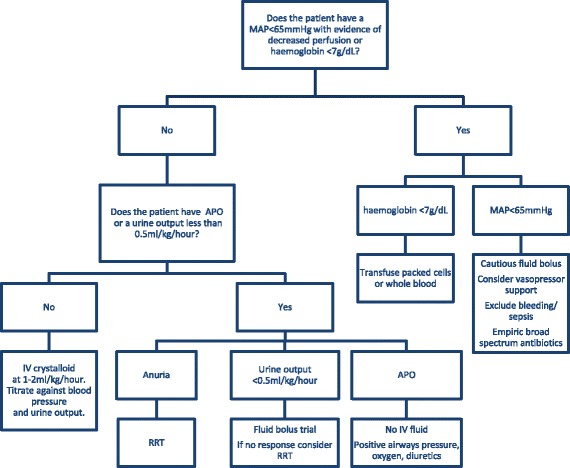
**Suggested fluid management for adults with severe malaria.** All of the proposed supportive care measures may not be available at sites where patients with severe malaria are initially managed. Early transfer to centers where these services are available is indicated, when possible. Maintenance fluid: the suggested 1 to 2 mL/kg per hour should take into consideration and include other administered fluids: antibiotic therapy, vasopressor infusions, and so on. Crystalloid: Based on plasma electrolytes, consider balanced solutions if available. Fluid bolus: 5 mL/kg crystalloid over 15 minutes; titrate bolus frequency against clinical response. APO, acute pulmonary edema; IV, intravenous; MAP, mean arterial pressure; RRT, renal replacement therapy, hemofiltration preferred if available.

There is no single fluid prescription appropriate for all adults with SFM. The disease’s protean manifestations occur in a range of patients with a variety of co-morbidities. However, in general, the available data support a conservative fluid resuscitation strategy (Figure 2). Although simple extrapolation to adults is not possible, children in the control arm of the FEAST study had the lowest mortality and received a cumulative median fluid load of only 10 mL/kg in the first 8 hours [51].

In the minority of adults with SFM in whom it occurs, shock remains a medical emergency, necessitating thorough assessment for concomitant bacterial infection (with initiation of broad-spectrum antibiotics) and exclusion of occult bleeding. In these patients, prompt fluid resuscitation with frequent reassessment remains the standard of care with early vasopressor support if required. Patients with a hemoglobin level of below 7 g/dL should receive fresh blood or packed cells. As anemia often evolves during treatment, clinicians should anticipate a transfusion requirement in those with more modest anemia.

If patients are anuric, fluid resuscitation is unlikely to lead to a recovery of renal function and early RRT is recommended. In the setting of AKI and oliguria, small fluid boluses (5 mL/kg titrated against effect) could be trialed, although clinicians should maintain a low threshold for initiating RRT. However, if an adult with SFM has adequate blood pressure (mean arterial blood pressure of more than 65 mm Hg) and urine output (more than 0.5 mL/kg per hour), there appears little advantage in prescribing any fluid beyond crystalloid at a maintenance rate of 1 to 2 mL/kg per hour, with the selection of crystalloid therapy guided by plasma electrolytes.

### Other malaria species

*Plasmodium vivax* and *Plasmodium knowlesi* can both cause severe disease, although sequestration is not prominent in either infection and the relative contribution of co-morbidities to clinical manifestations, particularly in vivax, is uncertain [73,74]. However, both infections can cause APO with a clinical phenotype similar to that seen in SFM. No studies of fluid resuscitation in patients with vivax or knowlesi infection have been performed, though given the risk of APO, a conservative fluid regimen with early RRT and vasopressor support is recommended.

## Conclusions

The optimization of volume status is a fundamental aspect of critical care, and liberal fluid resuscitation is life-saving in many conditions. However, in falciparum malaria, a disease with a unique pathophysiology, it is hazardous and fails to address the microvascular obstruction seen in the disease. Adults with SFM usually have an acceptable cardiac output and blood pressure and are frequently not volume-responsive; any extra administered fluid tends to accumulate in the interstitial space, potentially exacerbating the already impaired end-organ perfusion. Liberal fluid loading in adults leads to no meaningful improvement in the acidosis and AKI and increases the incidence of APO, and in children it increases mortality in the resource-poor setting. Despite the theoretical appeal of promptly correcting hypovolemia in adults with severe malaria, clinicians caring for patients should follow the old malariologist’s adage and ‘run them dry’.

## Additional file

Additional file 1:
**An orthogonal polarization spectral imaging video of a patient with severe falciparum malaria.**

## Abbreviations

AKI: Acute kidney injury
APO: Acute pulmonary edema
CVC: Central venous catheter
CVP: Central venous pressure
DO_2_: Oxygen delivery
EVLW: Extravascular lung water
FEAST: Fluid Expansion as Supportive Therapy
GEDVI: Global end-diastolic volume index
PaO_2_/FiO_2_: Arterial partial pressure of oxygen/fraction of inspired oxygen
PAOP: Pulmonary artery occlusion pressure
PD: Peritoneal dialysis
PRISM: PiCCO-guided Resuscitation in Severe Malaria
RRT: Renal replacement therapy
SFM: Severe falciparum malaria
VHS: Vietnamese Hemodynamic Study
WHO: World Health Organization
